# Supplementary material for: Microbiota-directed fibre activates both targeted and secondary metabolic shifts in the distal gut
Source: Nat Commun. 2020 Nov 13;11:5773. doi: 10.1038/s41467-020-19585-0 (PMC7666174; doi:10.1038/s41467-020-19585-0)
Supplement: Supplementary file 1 — Supplementary Information [file 41467_2020_19585_MOESM1_ESM.pdf]

SUPPLEMENTARY MATERIAL for:

Microbiota-directed fibre activates both targeted and secondary metabolic shifts in the distal gut

Leszek Michalak[1], John Christian Gaby[1], Leidy Lagos[2], Sabina Leanti La Rosa[1], Torgeir R. Hvidsten[1], Catherine Tétard-Jones[3], William G.T. Willats[3], Nicolas Terrapon[4,5], Vincent Lombard[4,5], Bernard Henrissat[4,5,6], Johannes Dröge[7], Magnus Øverlie Arntzen[1], Live Heldal Hagen[1], Margareth Øverland[2], **Phillip B. Pope[1,2]*, Bjørge Westereng[1]***

This PDF file includes:

I. Supplementary Figures and Legends
II. Supplementary Tables and Legends
III. References cited in the Supplementary Material

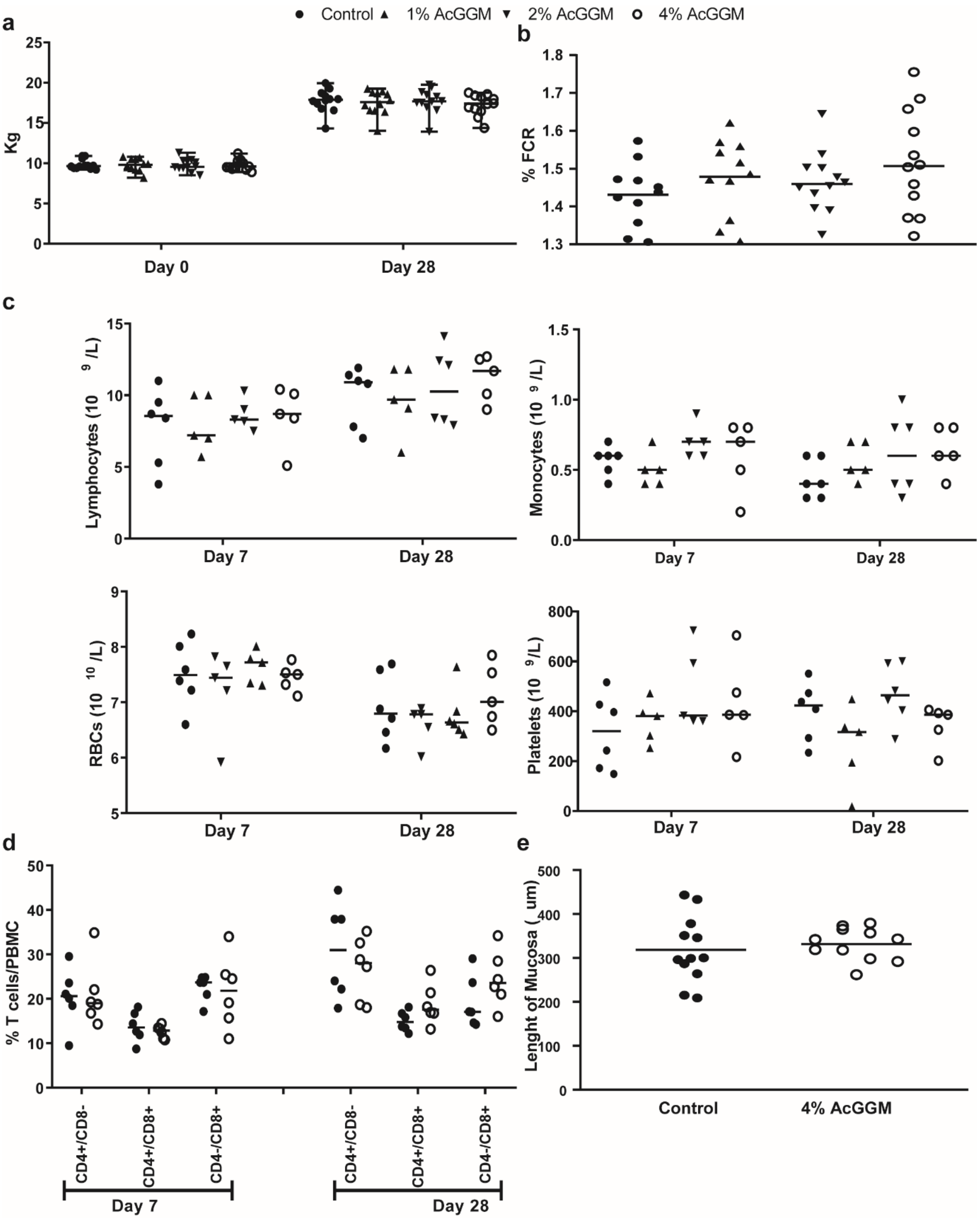

Supplementary Figure 1. Performance metrics and health status of weaning piglets subjected to various AcGGM-containing feeds. a. Average weight of piglets at the start of the feeding trial and at sampling day shows no statistically significant difference in Dunnett's multiple comparisons test (p=0.05). **b.** Feed conversion rate for piglets in the 4% AcGGM inclusion levels shows no statistically significant difference Dunnett's multiple comparisons test (p=0.05). **c.** Neither the numbers of lymphocytes, RBCs, monocytes nor platelets were significantly affected by the inclusion of AcGGM in diet (Tukey's multiple comparisons, p=0.05). **d.** Flow cytometry analysis of T cell populations in piglets from the control vs 4%

AcGGM inclusion group shows no statistically significant shift in any of the subpopulations (two-way ANOVA, p=0.05) (gating strategy: see Supplementary Figure 7). Y-axis - abundance of cells in percent of peripheral blood mononuclear cells. **e.** Colon epithelium morphology: length of mucosal layer shows no significant difference between the controls and 4% AcGGM inclusion. Length of mucosa was analyzed using Two-sample unpaired t-test (p=0.6) while the other parameters were analyzed using a nonparametric Wilcoxon test Numerical values shown in the charts are available in Supplementary Data 1 and Supplementary Table 1 (colon morphology).

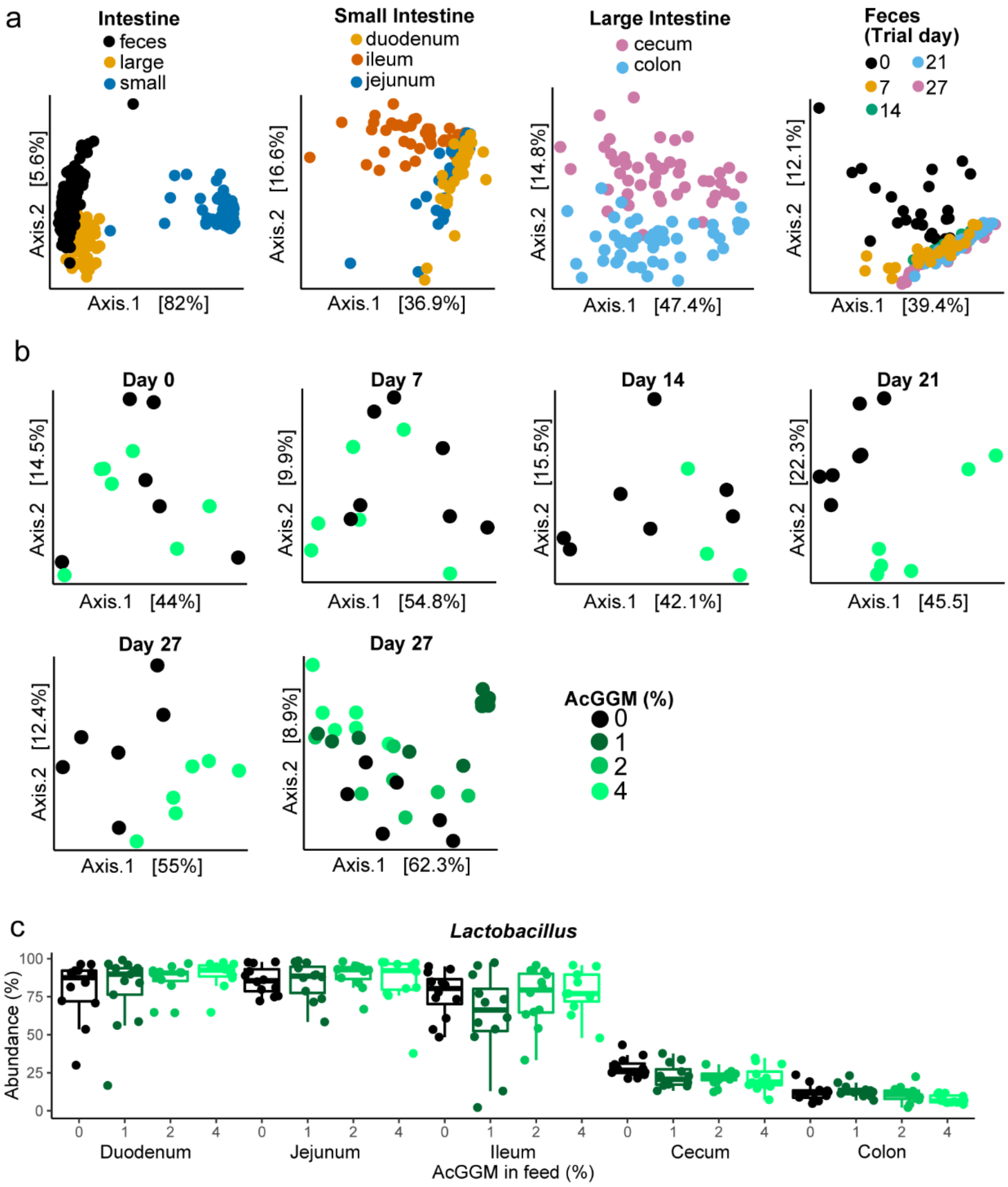

Supplementary Figure 2. Spatial and temporal changes in the gut microbiome of weaning piglets fed varying levels of AcGGM. Structural microbiome changes were monitored using DNA extracted from gut or fecal samples and 16S rRNA gene analysis over the month-long trial. **a.** Spatial variation of the gut microbiome of weaning piglets was observed between different regions within the small and large intestines as well as feces. **b.** Temporal microbiome changes of weaning piglets fed varying levels of AcGGM. Ordination plots of Bray-Curtis distances between microbiome communities analyzed from fecal samples collected from 6 randomly selected animals per treatment group, in weekly intervals, throughout the 28-day trial. From day 14 onwards, greater variation was observed between the control samples and the samples from the three AcGGM inclusion levels (1, 2 and 4%), indicating structural changes in the pig fecal microbiome composition in response to AcGGM inclusion. **c.** Spatial variation was observed for specific taxa such as the *Lactobacillus*, which was observed at higher relative abundances in regions within the small intestine. The boxes span the 25th–75th percentiles with the central bars being the medians. Whiskers extend maximum up to 1.5× the inter-quartile range (IQR) or, when all values are within 1.5× IQR, then the whisker extends to the most extreme data point.

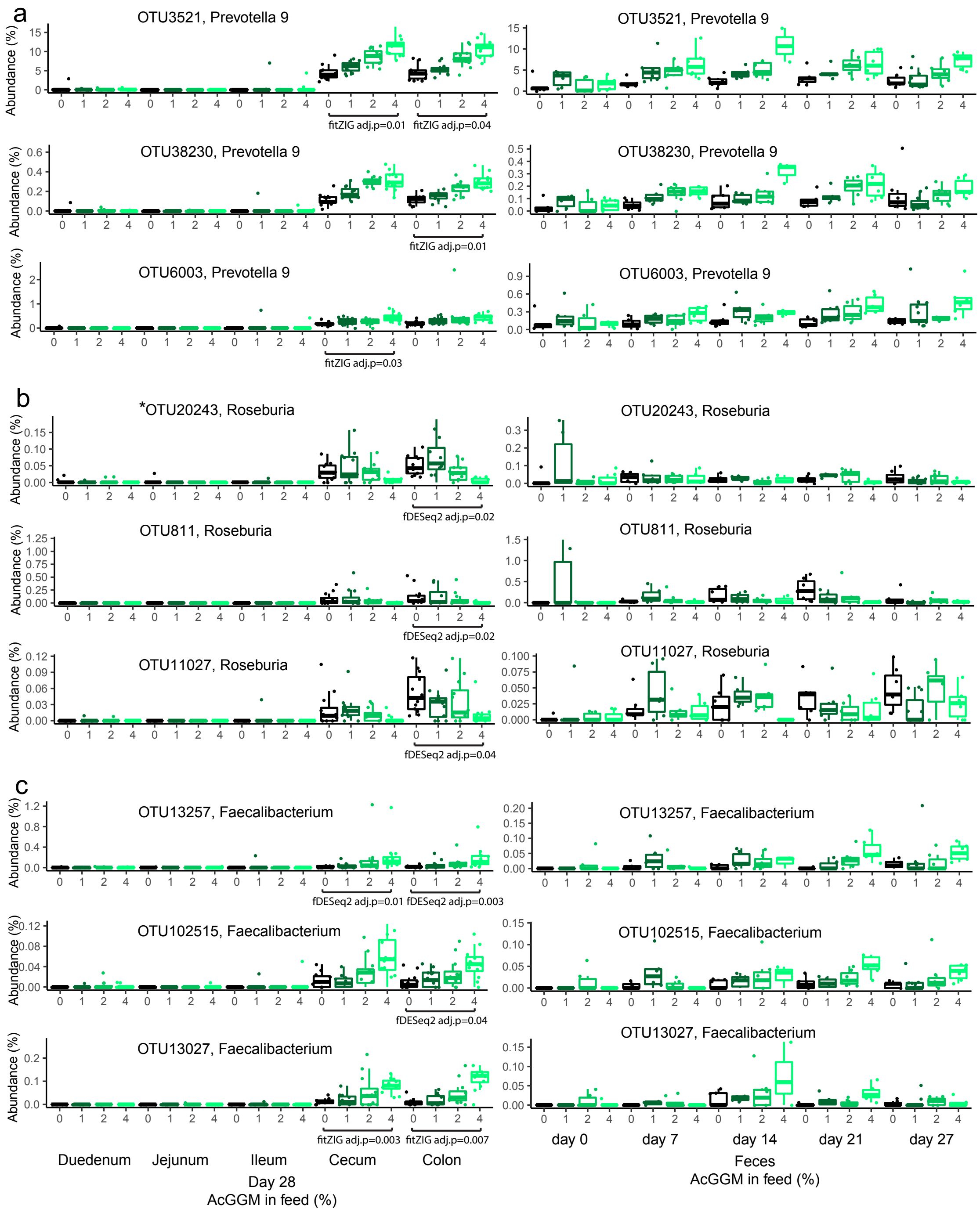

Supplementary Figure 3. Spatial and temporal changes of selected *Prevotella*- (a), *Roseburia*- (b) and *Faecalibacterium*- (c) affiliated phylotypes in fecal microbiome from weaning piglets fed varying levels of AcGGM. Differential abundance analysis was performed between animals being fed either the control or 4% AcGGM diets with the MetagenomeSeq fitZIG and DESeq2 negative binomial algorithms via the QIIME wrapper[1]. A complete list of OTU abundances that changed significantly (adjusted p<0.05) in response to AcGGM diets at day 28 are listed in Supplementary Data 2 and are denoted here in **a-c** when p<0.05. The boxes span the 25th–75th percentiles with the central bars being the medians. Whiskers extend maximum up to 1.5× the inter-quartile range (IQR) or, when all values are within 1.5× IQR, then the whisker extends to the most extreme data point.

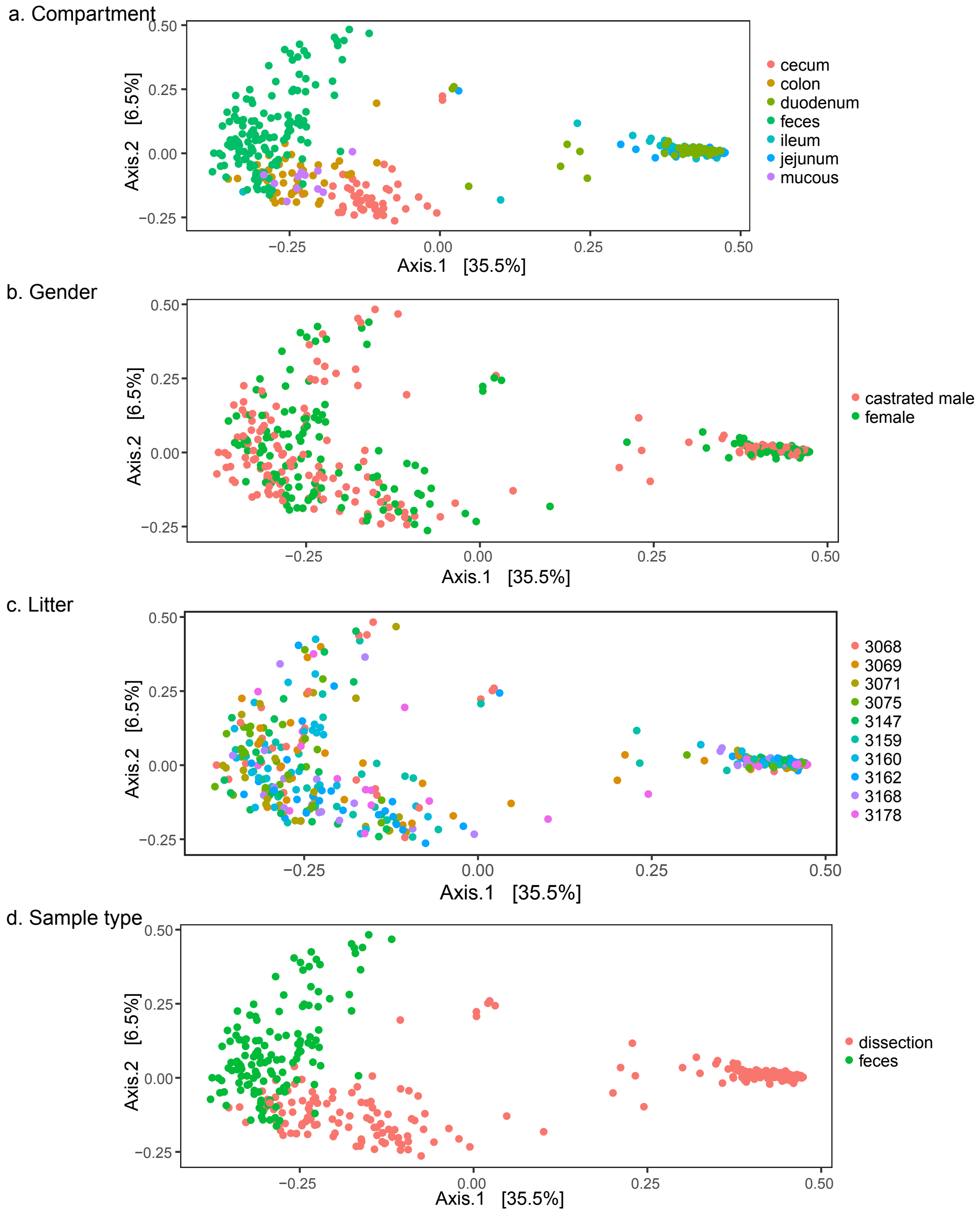

Supplementary Figure 4. Microbiota composition of samples from different sites in the pig gastrointestinal tract (GIT). Data is presented as a multidimensional scaling (MDS) ordination of weighted UNIFRAC distances. **a** Samples from all GIT compartments (including feces), were labelled according to GIT compartment of origin. The two clusters separate samples from upper GIT (duodenum, jejunum, ileum) in the right side of the plot, from fecal samples and lower GIT and (colon, cecum, colon mucus). No biases were observed from any of the variables (**b** Piglet gender. **c** Litter **d** Sample type) listed here.

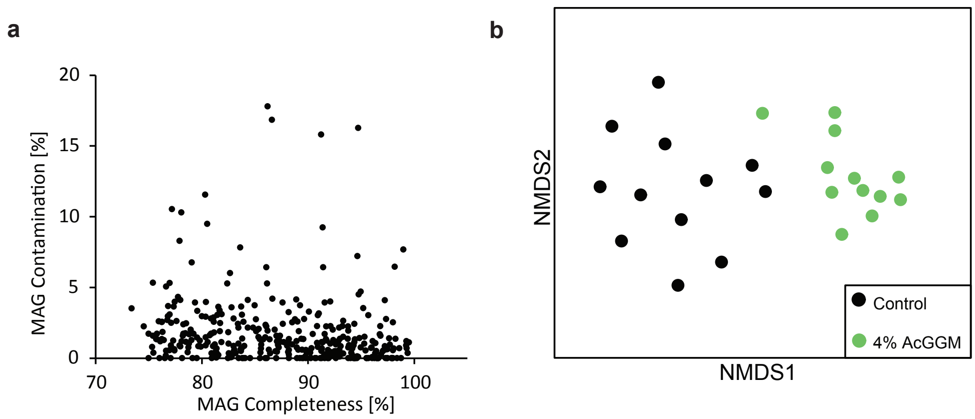

Supplementary Figure 5. **Metagenomic and metaproteomic analysis of the colon microbiome population of control and 4% AcGGM fed piglets. a.** Completeness % vs contamination % of the 355 MAGs recovered from 24 colon samples. Completeness and contamination was determined for each MAG using CheckM[2] version 1.0.7. A total of 145 had >90% completeness and were considered high quality according to the Genomics Consortium Standards[3] **b.** Non-metric multidimensional scaling (NMDS) ordination plot of sample distances calculated with MASH based analysis on the genetic content of MAGs recovered from metagenomes, sequenced and analyzed from colon samples collected from animals fed the control (n=12) and 4% AcGGM diets (n=12). Sample distribution shows the piglets from each dietary group had distinct genomic composition.

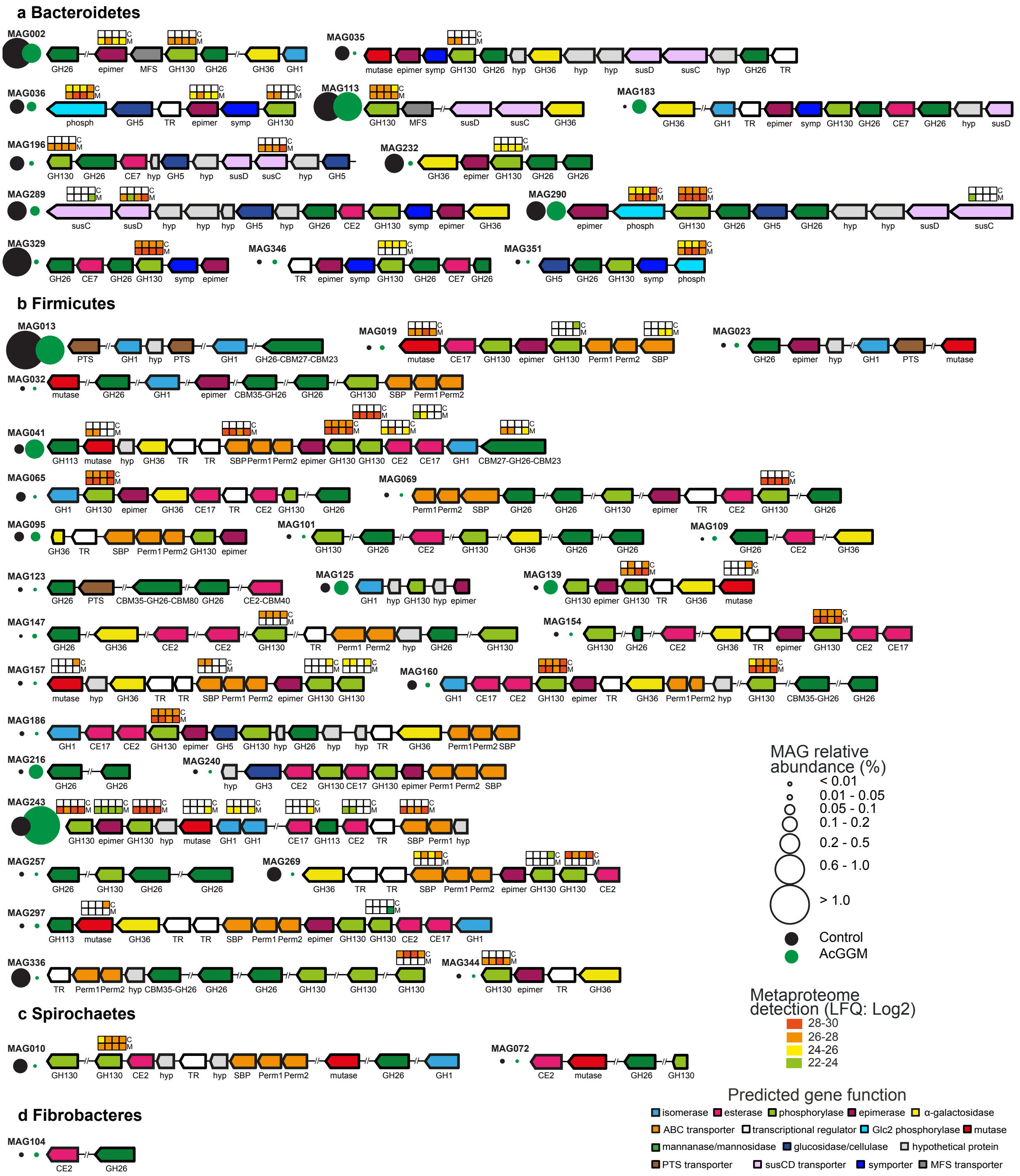

Supplementary Figure 6. Metaproteomic detection of predicted mannan-PULs encoded in Firmicutes-affiliated MAGs in pigs fed with the control or 4% AcGGM diet. Predicted PULs were determined using PULDB[4] (Bacteroidetes), which combines CAZymes[5], outer-membrane transport and carbohydrate-binding lipoproteins (SusC/D-like), as well as from previous biochemical and structural characterization of the mannan degradation cluster in *R. intestinalis* L1-82[6]. Clusters were affiliated to phyla: Bacteroidetes (**a**), Firmicutes (**b**), Spirochaetes (**c**) and Fibrobacteres (**d**). Heat maps above detected enzymes show the LFQ detection levels for the four replicates sampled in control (C) and 4% AcGGM-fed (M) pigs.

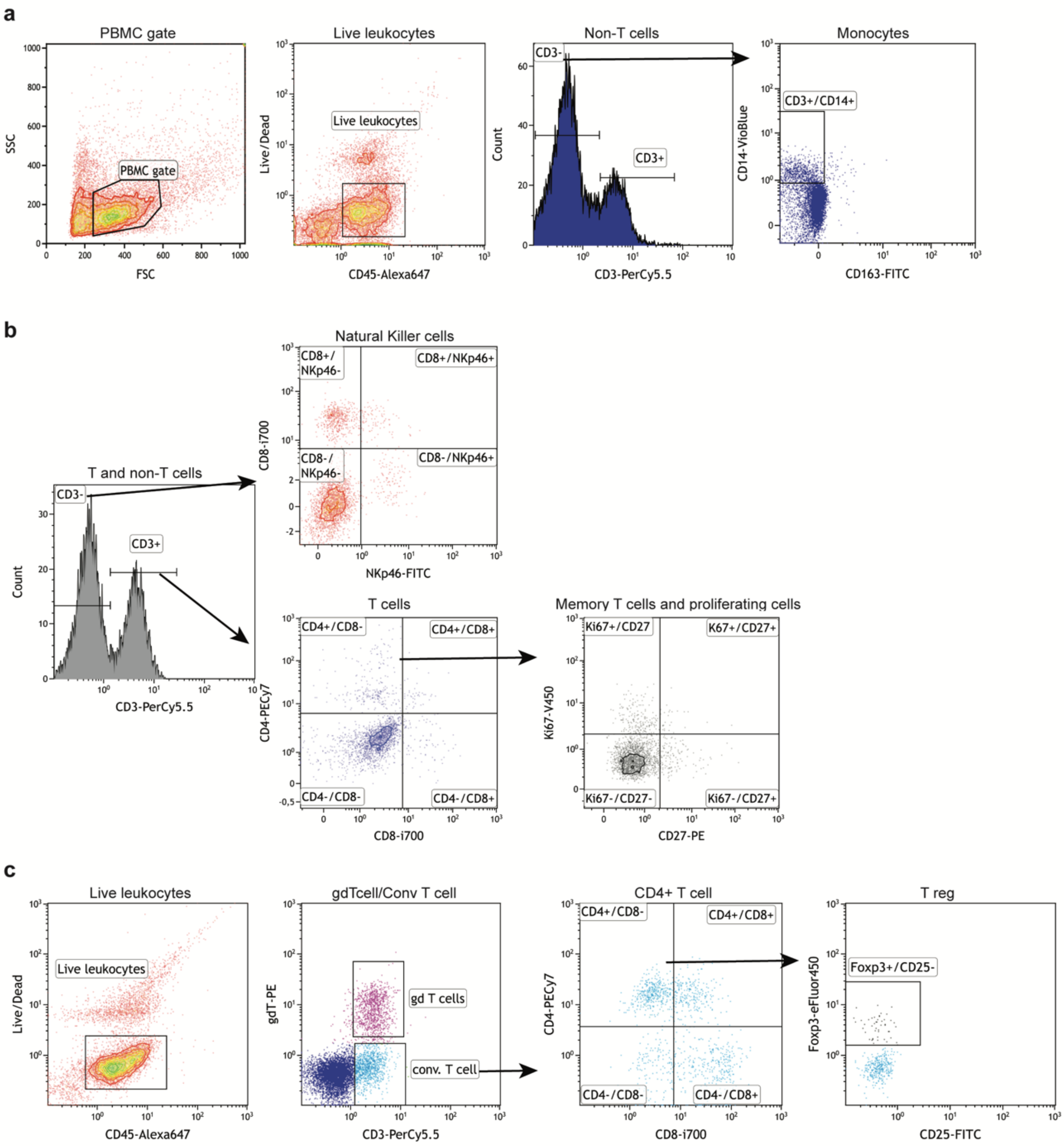

Supplementary Figure 7. Gating strategy used for flow cytometric data presented in Supplementary Figure 1. a. Cells were gated on SSC vs FSC to determine size and granularity, thereafter cells were gated based on live/dead to identify live leukocytes, which were used for further analysis. Monocytes were assigned as CD45+, CD3- and CD14+ or CD163+. **b.** Natural killer cells were assigned as CD45+, CD3- and CD8+ or NKp46+ while the different subsets of T cells were determined according to the expression of CD4, CD8, Ki67 and CD27. **c.** T reg were identified according to the expression of gdT (gdT cells) or CD3, CD4, CD8 (conventional T cells) or Foxp3 and CD25 (T reg). Supplementary Data 1 contains the percentage of each gate.

Supplementary Table 1. **Evaluation of colon morphology**. Analysis was performed according to the scoring system described by Fabia et al.[7] and indicates no significant difference between the control and 4% AcGGM inclusion level. Length of mucosa was analyzed using Two-sample unpaired t-test, p=0.6, while the other parameters were analyzed using a nonparametric Wilcoxon test.

	0% AcGGM	4% AcGGM
Ulceration	0	0
Mucosal atrophy	0	0
Edema	0	0
Cell infiltration	0.6	0.5
Vascular dilation	0.8	0.3
Length of mucosa (μm)	318±21.6	332±11.2

Supplementary Table 2. Total reads and sequence length obtained in the whole metagenome sequencing of each sample. 0M and 4M in “Sample id” designate 0% and 4% AcGGM content in diet.

Sample id	total reads per sample	total bases per sample	total Gbases per sample	Accession number (SRA)
**01_0M**	149875718	22481357700	22.48	SRR10209687
**02_0M**	39328376	5899256400	5.89	SRR10209686
**03_0M**	41490606	6223590900	6.22	SRR10209675
**04_0M**	50328468	7549270200	7.54	SRR10209670
**05_0M**	51520620	7728093000	7.72	SRR10209669
**06_0M**	44035782	6605367300	6.60	SRR10209668
**07_0M**	71111130	10666669500	10.66	SRR10209667
**08_0M**	38528542	5779281300	5.78	SRR10209666
**09_0M**	48341876	7251281400	7.25	SRR10209665
**10_0M**	43270632	6490594800	6.50	SRR10209664
**11_0M**	86191960	12928794000	12.93	SRR10209685
**12_0M**	60690558	9103583700	9.10	SRR10209684
		**0M total:**	**108.71**	
**13_4M**	176308520	26446278000	26.45	SRR10209683
**14_4M**	46804332	7020649800	7.02	SRR10209682
**15_4M**	53833158	8074973700	8.07	SRR10209681
**16_4M**	49041518	7356227700	7.35	SRR10209680
**17_4M**	61654754	9248213100	9.24	SRR10209679
**18_4M**	42908204	6436230600	6.43	SRR10209678
**19_4M**	34987350	5248102500	5.25	SRR10209677
**20_4M**	44732402	6709860300	6.71	SRR10209676
**21_4M**	33761876	5064281400	5.06	SRR10209674
**22_4M**	55012540	8251881000	8.25	SRR10209673
**23_4M**	45067490	6760123500	6.76	SRR10209672
**24_4M**	40208238	6031235700	6.03	SRR10209671
		**4M total:**	**102.65**	
		mean:	8.8	
		median:	7.1	
		max:	26.4	
		min:	5.1	

Supplementary Table 3. Composition and chemical content of basal diet.

Ingredients (%)		Calculated content (g/Kg)	
Wheat	51	Energy, MJ/kg	14.14
Barley	20	Dry Matter	884
Soybean meal	8	Crude protein	176.91
Oats	6	Crude fibre	34
Soy oil	4	Digestible crude protein	156.11
Fish meal	2	Starch	431.28
Potato protein	2	Crude fat	55
Corn gluten	1.78	Calcium	9.13
Calcium phosphate	1.13	Phosphorous	5.82
Limestone	1	Digestible phosphorous	3.73
Vitamin mix	1	Sodium	2.51
Lysine	0.69	Chloride	4.59
Salt	0.39	Lysine	13.04
Sodium bicarbonate	0.36	Digestible Lys	12.06
Threonine	0.25	Methionine + Cysteine	7.27
Methionine	0.15	Digestible Methionine + Cysteine	6.57
Valine	0.14	Methionine	4.21
Tryptophan	0.075	Threonine	8.11
		Tryptophan	2.70
		Valine	9.03

AcGGM was assigned net energy value zero.

*VilomixMineral premix and vitamin mineral premix provided the following per kilogram of diet: vitamin A, 12 000 IU; vitamin D_3, 3200 IU; vitamin E, 80 IU; vitamin K_3, 25 mg; vitamin B_1, 25 mg; vitamin B_2, 65 mg; vitamin B_6, 5 mg; vitamin B_{12}, 0.5 mg; niacin, 45 mg; pantothenic acid, 20 mg; folic acid 15 mg; biotin 0.15 mg. §Fe,150 mg; Cu, 125 mg; Zn, 150 mg; Mn, 30 mg; I, 0.3 mg; Se, 0.3 mg.

Supplementary Table 4. Primers used for 16S amplicon sequence data, for all fecal and intestinal samples.

Primers	Sequence
Pro341F	5'-CCT ACG GGN BGC ASC AG-3'
Pro805R	5'-GAC TAC NVG GGT ATC TAA TCC-3'

REFERENCES.

1 Love, M. I., Huber, W. & Anders, S. Moderated estimation of fold change and dispersion for RNA-seq data with DESeq2. *Genome Biol.* **15**, 550 (2014).

2 Parks, D. H., Imelfort, M., Skennerton, C. T., Hugenholtz, P. & Tyson, G. W. CheckM: assessing the quality of microbial genomes recovered from isolates, single cells, and metagenomes. *Genome Res* **25** (2015).

3 Bowers, R. M. *et al.* Minimum information about a single amplified genome (MISAG) and a metagenome-assembled genome (MIMAG) of bacteria and archaea. *Nat Biotechnol.* **35**, 725-731 (2017).

4 Terrapon, N., Lombard, V., Gilbert, H. J. & Henrissat, B. Automatic Prediction of Polysaccharide Utilization Loci in Bacteroidetes Species. *Bioinformatics* **31**, 647-655 (2014).

5 Martens, E. C., Koropatkin, N. M., Smith, T. J. & Gordon, J. I. Complex glycan catabolism by the human gut microbiota: the Bacteroidetes Sus-like paradigm. *J. Biol. Chem.* **284**, 24673-24677 (2009).

6 La Rosa, S. L. *et al.* The human gut Firmicute *Roseburia intestinalis* is a primary degrader of dietary β-mannans. *Nat. Commun.* **10**, 905 (2019).

7 Fabia, R. *et al.* The Effect of Exogenous Administration of Lactobacillus reuteri R2LC and Oat Fiber on Acetic Acid-Induced Colitis in the Rat. *Scandinavian Journal of Gastroenterology* **28**, 155-162 (1993).
