# Supplementary material for: Microbiota-directed fibre activates both targeted and secondary metabolic shifts in the distal gut
Source: Nat Commun. 2020 Nov 13;11:5773. doi: 10.1038/s41467-020-19585-0 (PMC7666174; doi:10.1038/s41467-020-19585-0)
Supplement: Supplementary file 3 — Description of Additional Supplementary Files [file 41467_2020_19585_MOESM3_ESM.docx]

**File Name:** Supplementary Data 1.

**Description:** Metadata recorded from weaning piglets subjected to various AcGGM-containing feeds. Measurements include: weight, feed conversion ratio, digesta pH, flow cytometry, hematology and fecal score. Weight, Feed conversion ratio and digesta pH were measured for the 48 piglets, while flow cytometry was performed on 12 animals, 6 piglets control and 6 piglets 4% AcGGM. Hematology measurements were determinate for 24 animals, 6 piglets per diet. Fecal scores were determined daily per pen and were quantified according to firmness and shape as described in Methods, with score 1 = firm and shaped and score = 4 watery. Samples with score 3 or 4 are considered diarrheic. SCFA content of digesta samples from cecum and colon was analyzed on an RSLC Ultimate 3000 (Dionex, USA) HPLC using a REZEX ROA-Organic Acid H+ 300x7.8mm ion exclusion column (Phenomenex, USA) at 65°C, 10 µL injection volume, with isocratic elution using 0.6 mL/min of 5mM H_2_SO_4_ as mobile phase and a UV detector set to 210 nm. The SCFAs were recorded as millimolar concentration in the digesta and analyzed using a two-tailed *t* test.

**File Name:** Supplementary Data 2.

**Description:** Relative abundance of operational taxonomic units that were observed to change in response to one month of the host animals being fed varying AcGGM inclusion levels. Samples from the cecum and colon were collected at day 28 and analyzed using 16S rRNA gene sequencing analysis. Differential abundance analysis was performed between animals being fed either the control or 4% AcGGM diets with the MetagenomeSeq fitZIG and DESeq2^1^ negative binomial algorithms via the QIIME wrapper. OTUs with adjusted p-values <0.05 are listed.

**File Name:** Supplementary Data 3.

**Description:** Relative abundance and taxonomic affiliation of MAGs reconstructed from the colon of pigs fed either the control or 4% AcGGM diet. Taxonomic classification (determined via GTDB-Tk) is given for 355 MAGs, while their relative abundance (determined via CoverM) across metagenomes generated for 12 x control pigs (01_0M-12_0M) and 12 x 4% AcGGM pigs (13_4M-24_4M) are listed and analyzed using a two-tailed *t* test.

**File Name:** Supplementary Data 4.

**Description:** Concatenated ribosomal protein tree of 22 ribosomal proteins for all MAGs in the pig distal gut microbiome, with reference sequences in Newick format.

**File Name:** Supplementary Data 5.

**Description:** Total counts, means and standard deviation of detected proteins mapped to MAGs analyzed in weaning piglets fed either the control or 4% AcGGM diets. Four randomly selected colon samples from animals fed either the control (C) or mannan (M) diet were selected for metaproteomic analysis, which detected a total of 8515 protein groups that mapped against 355 MAGs reconstructed from the colon.

**File Name:** Supplementary Data 6.

**Description:** Metabolic reconstruction for selected MAGs and LFQ intensities of their detected proteins in samples analyzed from weaning piglets fed either the control (C) or 4% AcGGM (M) diet. Key metabolic enzymes and pathways are annotated for each ORF within each MAG (E.C. and CAZy family where available) as well as gene names are provided. Number and gene names in square parenthesis indicate enzymes contributing to metabolic pathways that are illustrated in Fig. 7. Pathway reconstruction is provided for predicted butryrate-producers (MAG041, MAG243, MAG133, MAG269, MAG292, MAG324), *Prevotella*-affiliated populations (MAG191, MAG285, MAG196, MAG034) and other populations whose proteomes were highly detected/enriched in our analysis (MAG053, MAG150, MAG013, MAG225, MAG048). *The complete E.C. list for each CAZy family can be found at CAZy.org. In many instances, multiple E.C. numbers are listed for each CAZy family and are constantly being upgraded as more biochemical information becomes available.

**File Name:** Supplementary Data 7.

**Description:** MAG enrichment analysis. MAGs enriched in hierarchal metaproteome expression clusters (see Fig. 4c), determined using 4562 unique groups (consisting of 12 535 shared proteins). That is, MAGs that contribute with more detected proteins in a cluster than what we would expect by chance. Five different clusters were observed, with protein groups differentially detected in AcGGM fed pigs (M1-4), control pigs (C1-4), all pigs (M1-4 + C1-4), AcGGM fed pigs plus one control (M1-4 + C4) and only in individual pigs (Individual). MAGs in each cluster are ranked by adjusted p-value. x denotes total number of detected (shared) proteins for a given MAG within a given expression cluster. k denotes total number of detected (shared) proteins within a given expression cluster. m denotes total number of detected (shared) proteins within a given MAG. N denotes total number of detected (shared) proteins in the complete dataset. MAG enrichment analysis was performed using the hypergeometric distribution function phyper in R with the false discovery rate controlled at 5% using the function p.adjust with method = "BH"

**File Name:** Supplementary Data 8.

**Description:** MAPP analysis. Micro Array Polymer Profiling (MAPP) of plant cell wall components in each diet prior to feeding as well as colon samples. Colour intensity is proportional to mean spot signal. Starch was the most detected fibre in the basal feed and was readily digested in pigs fed either the control or AcGGM diets. Mannan fibres in both the basal feed as well as AcGGM were not detected, presumably due to the lack of specificity between the *Thermotoga maritima* CBM27 probe (Genbank: AAD36302) and/or the ineffectiveness of the extraction method to dissociate mannan fibres from other plant cell wall fibres in order for them to be detected. Xylan and xyloglucan fibres were observed to increase in digesta samples, presumably due to the deconstruction of plant cell walls and the liberation of these fibres.

**File Name:** Supplementary Data 9.

**Description:** EC enrichment analysis. EC-annotations enriched in hierarchal metaproteome expression clusters (see Fig. 4c), determined using 4562 unique groups (consisting of 12 535 shared proteins). That is, designated EC annotations that contribute with more detected proteins in a cluster than what we would expect by chance. Five different clusters were observed, with protein groups differentially detected in AcGGM fed pigs (M1-4), control pigs (C1-4), all pigs (M1-4 + C1-4), AcGGM fed pigs plus one control (M1-4 + C4) and only in individual pigs (Individual). EC-annotations in each cluster are ranked by adjusted p-value. **x** denotes total number of detected protein groups for a given EC annotation number within a given expression cluster. **k** denotes total number of detected (shared) proteins within a given expression cluster with an EC annotation number. **m** denotes total number of detected protein groups with a given EC annotation number. **N** denotes total number of detected protein groups in the complete dataset with an EC annotation number. EC enrichment analysis was performed using the hypergeometric distribution function phyper in R with the false discovery rate controlled at 5% using the function p.adjust with method = "BH
