# Supplementary material for: Microbiota-directed fibre activates both targeted and secondary metabolic shifts in the distal gut
Source: Nat Commun. 2020 Nov 13;11:5773. doi: 10.1038/s41467-020-19585-0 (PMC7666174; doi:10.1038/s41467-020-19585-0)
Supplement: Supplementary file 13 — Reporting Summary [file 41467_2020_19585_MOESM13_ESM.pdf]

nature research

Corresponding author(s): Phil B. Pope
Bjørge Westereng
J. Chris Gaby

Last updated by author(s): Oct 12, 2020

Reporting Summary

Nature Research wishes to improve the reproducibility of the work that we publish. This form provides structure for consistency and transparency in reporting. For further information on Nature Research policies, see Authors & Referees and the Editorial Policy Checklist .

Statistics

For all statistical analyses, confirm that the following items are present in the figure legend, table legend, main text, or Methods section.

n/a	Confirmed	
☐	☒	The exact sample size (*n*) for each experimental group/condition, given as a discrete number and unit of measurement
☐	☒	A statement on whether measurements were taken from distinct samples or whether the same sample was measured repeatedly
☐	☒	The statistical test(s) used AND whether they are one- or two-sided<br>*Only common tests should be described solely by name; describe more complex techniques in the Methods section.*
☐	☒	A description of all covariates tested
☐	☒	A description of any assumptions or corrections, such as tests of normality and adjustment for multiple comparisons
☐	☒	A full description of the statistical parameters including central tendency (e.g. means) or other basic estimates (e.g. regression coefficient) AND variation (e.g. standard deviation) or associated estimates of uncertainty (e.g. confidence intervals)
☐	☒	For null hypothesis testing, the test statistic (e.g. *F*, *t*, *r*) with confidence intervals, effect sizes, degrees of freedom and *P* value noted<br>*Give P values as exact values whenever suitable.*
☒	☐	For Bayesian analysis, information on the choice of priors and Markov chain Monte Carlo settings
☒	☐	For hierarchical and complex designs, identification of the appropriate level for tests and full reporting of outcomes
☒	☐	Estimates of effect sizes (e.g. Cohen's *d*, Pearson's *r*), indicating how they were calculated

Our web collection on statistics for biologists contains articles on many of the points above.

Software and code

Policy information about availability of computer code

Data collection	No software was used for data collection
Data analysis	For flow cytometry analysis, data was processed using Kaluza 1.5 software (both Beckman Coulter, Inc.).<br><br>Processing of 16S rRNA gene amplicon data was done with a combination of standalone programs, QIIME version 1.9.1, RINSEQ version 0.20.4, VSEARCH version 2.3.2, Trimmomatic version 0.36, MOTHUR version 1.36.1 and the R package Phyloseq version 1.22.3.<br><br>For shotgun metagenomics, individual sample assembly was accomplished with metaSPAdes version 3.11.1. MegaHIT version 1.1.3 was used for co-assembly of all 24 samples together as well as co-assembly of the 12 control samples together and the 12 4% AcGGM samples together. MetaBAT version 0.26.3 was used to bin the assemblies, and dRep version 2.0.5 was used to dereplicate the multiple assembly and binning combinations to produce an optimal set of MAGs. MASH version 2.0 used to compare the similarity of the 24 metagenomes by calculating pairwise Jaccard distances which were imported into R version 3.6.0 for NMDS ordination and visualization. Completeness and contamination was determined for each MAG using CheckM version 1.0.7. Feature and functional annotation were completed with the Prokka pipeline version 1.12, and the predicted protein sequences from all 355 MAGs were concatenated to create the metaproteomics reference database. Resulting annotated open reading frames (ORFs) were retrieved, further annotated for CAZymes using the CAZy annotation pipeline with libraries from the July 2018 database release.<br><br>For metaproteomics, raw data was analyzed using MaxQuant version 1.4.1.2. The output from MaxQuant was further explored in Perseus version 1.6.0.7.<br><br>The code used to perform the metaproteomic enrichment analysis is available at: https://gitlab.com/hvidsten-lab/michalak

For manuscripts utilizing custom algorithms or software that are central to the research but not yet described in published literature, software must be made available to editors/reviewers. We strongly encourage code deposition in a community repository (e.g. GitHub). See the Nature Research guidelines for submitting code & software for further information.

Data

Policy information about availability of data

All manuscripts must include a data availability statement. This statement should provide the following information, where applicable:

- Accession codes, unique identifiers, or web links for publicly available datasets
- A list of figures that have associated raw data
- A description of any restrictions on data availability

The raw data of each piglet from the AcGGM-feeding experiments plotted in Fig S1 and have been reported in Supplementary Table 1 and Supplementary Dataset 1. Additional data is available from the corresponding authors on request.

All sequencing reads have been deposited at the NCBI sequence read archive under BioProject PRJNA574295 https://www.ncbi.nlm.nih.gov/bioproject/PRJNA574295, with specific numbers listed in Supplementary Table 2. All annotated MAGs are publicly available via doi.org/10.6084/m9.figshare.9816581. The proteomics data has been deposited to the ProteomeXchange Consortium (http://proteomecentral.proteomexchange.org) via the PRIDE partner repository30 with the dataset identifier PXD015757 https://www.ebi.ac.uk/pride/archive/projects/PXD015757.

Field-specific reporting

Please select the one below that is the best fit for your research. If you are not sure, read the appropriate sections before making your selection.

- [x] Life sciences
- [ ] Behavioural & social sciences
- [ ] Ecological, evolutionary & environmental sciences

For a reference copy of the document with all sections, see nature.com/documents/nr-reporting-summary-flat.pdf

Life sciences study design

All studies must disclose on these points even when the disclosure is negative.

Sample size

For the dietary intervention experiments, Forty-eight piglets were divided into four treatment groups of twelve, housed in pens of four piglets. This design was selected to maximize the statistical power and minimize bias originating from individual variability. Number of piglets was selected based on the capacity of our facilities for endpoint sampling to ensure maximum quality of sample material. Our previous experiments on pig gut microbiome yielded reliable statistical results in animal sets as small as n=3 (PMID: 25905018) or n=10 (https://doi.org/10.1016/j.livsci.2007.02.002) per treatment group. Sample sizes, animal care protocols and experimental procedures were approved by the Norwegian Animal Research Authority, approval no. 17/9496, FOTS ID 11314 and treated according to institutional guidelines.

Data exclusions

No data was excluded from the data analysis.

Replication

The authors believe that within the constraints of working with large animals, the conclusions in the manuscript are supported with sufficient replicates and sample analysis as well as cross-referencing datasets (i.e. 16S rRNA gene analysis, metaproteomics). All technical and biological replicates are indicated in the respective figure legends.

Randomization

The piglets were randomly distributed into twelve groups (pens) of four animals, which was baseline normalized over litter, piglet weight and sex.

For metaproteomic analysis, a randomization function in the software R was used to randomly select four samples (from a total of twelve), each from the control group of pigs and those fed 4% AcGGM.

Blinding

The investigators were blinded to the group allocation during sample collection, processing and analysis.

Reporting for specific materials, systems and methods

We require information from authors about some types of materials, experimental systems and methods used in many studies. Here, indicate whether each material, system or method listed is relevant to your study. If you are not sure if a list item applies to your research, read the appropriate section before selecting a response.

Materials & experimental systems

n/a	Involved in the study	
[ ]	[x]	Antibodies
[x]	[ ]	Eukaryotic cell lines
[x]	[ ]	Palaeontology
[ ]	[x]	Animals and other organisms
[x]	[ ]	Human research participants
[x]	[ ]	Clinical data

Methods

n/a	Involved in the study	
[x]	[ ]	ChIP-seq
[ ]	[x]	Flow cytometry
[x]	[ ]	MRI-based neuroimaging

Antibodies

Antibodies used

Isolated PBMCs were incubated with Fixable Yellow Dead Cell Stain Kit (Life Technologies, Thermo Fisher Scientific Inc.) followed by primary monoclonal antibodies (mAbs), brief incubation with 30% normal pig serum to block Fc-receptors, and finally fluorescence-labeled secondary antibodies (Abcam plc, UK). Defined markers were used to identify the different immune subpopulations. For monocytes, the following antibodies were used:

CD45, (Mouse anti Pig CD45 antibody clone K252.1E4, Bio-rad, catalog no. MCA1222GA; dilution 1:100)

CD3, (Mouse anti Pig CD3 clone BB23-8E6-8C8, BD, catalog no. 561478; dilution 1:200)

CD14, (Anti-CD14 antibody (ab231049), Miltenyi Biotech, catalog no 130-113-714; dilution 1:20)

CD163, (Mouse anti Pig CD163 antibody clone 2A10/11, LSBio, catalog no. LS-C188273-0.1; dilution: 0.1 ul per 1 million cells in residual volume)

MHCII, (Mouse anti Pig SLA Class II DR antibody clone 2E9/13, Bio-rad, catalog no. MCA2314; dilution 1:100)

To analyze regulatory T cells (T reg) the following antibodies were used:
CD45, (Mouse anti Pig CD45 antibody clone K252.1E4, Bio-rad, catalog no. MCA1222GA; dilution 1: 100)

CD3, (Mouse anti Pig CD3 clone BB23-8E6-8C8; BD, catalog no. 561478; dilution 1:200)

TCR γ/δ, (APC Rat Anti-Pig γδ T Clone MAC320 (RUO), BD, catalog no. 561482; dilution 1:100)

CD4, (Mouse Anti-Pig CD4 clone 74-12-4, BD, catalog no. 561473, dilution 1:100)

CD8, (FITC Mouse Anti-Pig CD8a Clone 76-2-11 (RUO), SouthernBiotech, catalog no. 4520-08; dilution 1:400)

FOXp3 (FOXP3 Monoclonal Antibody (FJK-16s), ThermoFisher, catalog no. 14-5773-82; dilution 1:100)

CD25, (CD25 antibody (P4A10), ThermoFisher, catalog no. 11-0250-42; dilution 1:100)

To identify T and NK cells the following antibodies were used:
CD45, (Mouse anti Pig CD45 antibody clone K252.1E4, Bio-rad, catalog no. MCA1222GA; dilution 1:100)

CD8, (FITC Mouse Anti-Pig CD8a Clone 76-2-11 (RUO), SouthernBiotech, catalog no. 4520-08; dilution 1:400)

NKp46, (Mouse anti Pig CD335 antibody clone VIV-KM1, Bio-rad, catalog no. MCA5972; dilution 1:100)

CD4, (Mouse Anti-Pig CD4 clone 74-12-4, BD, catalog no. 561473, dilution 1:100)

Ki67, (Anti-Ki67 antibody (MKI67), BD, catalog no. 561281, dilution 1:100)

CD27, (Mouse anti Pig CD27 Antibody clone B30C7, Bio-rad, catalog no. MCA5973; dilution 1:100)

For MAPP analysis the following molecular probes were used:

LM, and JIM series: LM20 (epitope: HG partially esterified), JIM7 (epitope: HG partially esterified), LM19 (epitope: HG partially de-esterified), LM12 (epitope: Feruloylate on any polymer), LM6 (epitope: (1-5)-α-L-arabinan), LM13 (epitope: linearised (1-5)-α-L-arabinan), LM5 (epitope: (1-4)-β-D-galactan), LM22 (epitope: (1-4)-β-D-(gluco)mannan), LM21 (epitope: (1-4)-β-D-(galacto)(gluco)mannan), LM25 (epitope: xyloglucan / unsubstituted β-D-glucan), LM15 (epitope: xyloglucan (XXXG motif), LM10 (epitope: (1-4)-β-D-xylan), LM27 (epitope: Anti-grass xylan), LM28 (epitope: Glucuronoxylan), LM11 (epitope: 1-4)-β-D-xylan/arabinoxylan), JIM8 (epitope: arabinogalactan protein, AGP), JIM13 (epitope: AGP), LM2 (epitope: AGP), LM14 (epitope: AGP), JIM16 (epitope: AGP), LM1 (epitope: extensin), JIM12 (epitope: extensin), JIM20 (epitope: extensin), JIM11 (epitope: extensin); Plant Probes; dilution: 1 in 10 in TBS containing 5% milk protein. Secondary antibody: Goat Anti-Rat IgG Antibody, Alkaline Phosphatase conjugate, Sigma, catalog no. AP136A; dilution 1:1000 in TBS containing 5% milk protein.

BAM7: Sea Probes; dilution: 1 in 10 in TBS containing 5% milk protein. Secondary antibody: Goat Anti-Rat IgG Antibody, Alkaline Phosphatase conjugate, Sigma, catalog no. AP136A; dilution 1:1000 in TBS containing 5% milk protein.

BS Mannan, and MLG (mixed linkage glucan): BioSupplies; catalog no. 400-4: epitope (1-4)-B-mannan and galacto-(1-4)-B-mannan, 400-3: epitope (1-3,1-4)-B-glucan; dilution: 1 in 50 in TBS (tris buffered saline) containing 5% milk protein. Secondary antibody: Goat Anti-Mouse IgG Antibody, Alkaline Phosphatase conjugate, Sigma, catalog no. AP124A; dilution 1:1000 in TBS containing 5% milk protein.

INCh1, epitope: α-(1-4)-glucan; produced by and sourced from collaborators at INRAE (Institut National de la Recherche Agronomique); dilution 1 in 10 in TBS (tris buffered saline) containing 5% milk protein. Secondary antibody: Goat Anti-Mouse IgG Antibody, Alkaline Phosphatase conjugate, Sigma, catalog no. AP124A; dilution 1:1000 in TBS containing 5% milk protein.

Carbohydrate binding module 27A (CBM27A), epitope: 1,4-β-mannans; NZYtech, catalog no. CZ0475; dilution 1 in 50 in TBS (tris buffered saline) containing 5% milk protein. Secondary antibody: anti-polyHistidine-alkaline phosphatase antibody produced in mouse, Sigma, catalog no. A5588; dilution 1 in 1000 in TBS containing 5% milk protein.

Validation

All listed antibodies are commercially available and validated by the vendor for the assay described in this paper. Validation information for each antibody is available on the vendor website following the link provided below:

CD45: https://www.bio-rad-antibodies.com/monoclonal/pig-porcine-cd45-antibody-k252-1e4-mca1222.html?f=purified

CD3: https://www.bdbiosciences.com/eu/reagents/research/antibodies-buffers/immunology-reagents/other-species/cell-surface-antigens/percp-cy55-mouse-anti-pig-cd3-bb23-8e6-8c8/p/561478

CD14: https://www.miltenyibiotec.com/IT-en/products/macs-flow-cytometry/antibodies/primary-antibodies/cd14-antibodies-human-tuk4-1-50.html#vioblue:for-30-tests

CD163: https://www.lsbio.com/antibodies/anti-cd163-antibody-clone-2a10-11-fitc-ihc-if-immunofluorescence-wb-western-ip-flow-ls-c188273/196176

MHCII: https://www.bio-rad-antibodies.com/monoclonal/pig-porcine-sla-class-ii-dr-antibody-2e9-13-mca2314.html?f=fitc

To analyze regulatory T cells (T reg) the following antibodies were used:
CD45: https://www.bio-rad-antibodies.com/monoclonal/pig-porcine-cd45-antibody-k252-1e4-mca1222.html?f=purified

CD3: https://www.bdbiosciences.com/eu/reagents/research/antibodies-buffers/immunology-reagents/other-species/cell-surface-antigens/percp-cy55-mouse-anti-pig-cd3-bb23-8e6-8c8/p/561478

TCR γ/δ: https://www.bdbiosciences.com/eu/reagents/research/antibodies-buffers/immunology-reagents/other-species/cell-surface-antigens/apc-rat-anti-pig-t-lymphocytes-mac320/p/561482

CD4: https://www.bdbiosciences.com/eu/reagents/research/antibodies-buffers/immunology-reagents/other-species/cell-surface-antigens/pe-cy7-mouse-anti-pig-cd4a-74-12-4/p/561473

CD8: https://www.southernbiotech.com/?catno=4520-08&type=Monoclonal#&panel2-1

FOXp3: https://www.thermofisher.com/antibody/product/FOXP3-Antibody-clone-FJK-16s-Monoclonal/14-5773-82

CD25: https://www.thermofisher.com/antibody/product/CD25-Antibody-clone-P4A10-Monoclonal/11-0250-42

To identify T and NK cells the following antibodies were used:
CD45: https://www.bio-rad-antibodies.com/monoclonal/pig-porcine-cd45-antibody-k252-1e4-mca1222.html?f=purified

CD8: https://www.southernbiotech.com/?catno=4520-08&type=Monoclonal#&panel2-1

NKp46: https://www.bio-rad-antibodies.com/monoclonal/pig-porcine-cd335-antibody-viv-km1-mca5972.html?f=purified

CD4: https://www.bdbiosciences.com/eu/reagents/research/antibodies-buffers/immunology-reagents/other-species/cell-surface-antigens/pe-cy7-mouse-anti-pig-cd4a-74-12-4/p/561473

Ki67: https://www.bdbiosciences.com/us/applications/research/intracellular-flow/intracellular-antibodies-and-isotype-controls/anti-human-antibodies/v450-mouse-anti-ki-67-b56/p/561281

CD27: https://www.bio-rad-antibodies.com/monoclonal/pig-porcine-cd27-antibody-b30c7-mca5973.html?f=purified

For MAPP analysis
LM, and JIM series: http://www.plantprobes.net/index.php

BAM7: http://www.sb-roscoff.fr/en/seaprobes

BS Mannan and MLG: http://www.biosupplies.com.au/index.php?q=products.html

INCh1: laura.linxe@inrae.fr; Rydahl et al., 2017, Nat. Sci. Rep., 7, 9326 ; https://www.nature.com/articles/s41598-017-04307-2

CBM27A: https://www.nzytech.com/products-services/carbohydrate-binding-module-carbohydrate-binding-module/cz0475/?

sf_paged=63

Goat Anti-Rat IgG Antibody, Alkaline Phosphatase conjugate: https://www.sigmaaldrich.com/catalog/product/mm/ap136a?lang=en&region=GB

Goat Anti-Mouse IgG Antibody, Alkaline Phosphatase conjugate: https://www.sigmaaldrich.com/catalog/product/mm/ap124a?lang=en&region=GB

Anti-polyHistidine-alkaline phosphatase antibody produced in mouse: https://www.sigmaaldrich.com/catalog/product/sigma/a5588?lang=en&region=GB

Animals and other organisms

Policy information about studies involving animals; ARRIVE guidelines recommended for reporting animal research

Laboratory animals	A total of 48 cross bred piglets (Landrace x Yorkshire), 24 male and 24 female, with an average initial body weight (BW) of 9,8 ± 0,5 kg, weaned at 28 days of age were used for animal experiments
Wild animals	Study did not involve wild animals
Field-collected samples	Study did not involve field-collected samples
Ethics oversight	Animal care protocols and experimental procedures were approved by the Norwegian Animal Research Authority, approval no. 17/9496, FOTS ID 11314 and treated according to institutional guidelines.

Note that full information on the approval of the study protocol must also be provided in the manuscript.

Flow Cytometry

Plots

Confirm that:

- [x] The axis labels state the marker and fluorochrome used (e.g. CD4-FITC).
- [x] The axis scales are clearly visible. Include numbers along axes only for bottom left plot of group (a 'group' is an analysis of identical markers).
- [x] All plots are contour plots with outliers or pseudocolor plots.
- [x] A numerical value for number of cells or percentage (with statistics) is provided.

Methodology

Sample preparation	For flow cytometry analysis, whole blood was diluted 1:1 in RPMI 1640 and kept on ice until single cells isolation. For the isolation of peripheral blood mononuclear cells (PBMCs) blood was purified by centrifugation in a Ficoll gradient (Kreuzer et al. 2012). Then, isolated PBMCs were incubated with Fixable Yellow Dead Cell Stain Kit (Life Technologies, Thermo Fisher Scientific Inc.) followed by primary monoclonal antibodies (mAbs), brief incubation with 30% normal pig serum to block Fc-receptors, and finally fluorescence-labeled secondary antibodies (Abcam plc, UK). To detect the intracellular CD3 epitope, surface-labeled cells were permeabilized with Intracellular Fixation and Permeabilization Buffer Set (eBioscience, Affymetrix Inc.) according to the manufacturer's instructions. Labeled cells were analyzed on a Gallios Flow Cytometer (Beckman Coulter, Inc.) and data were processed using Kaluza 1.5 software (both Beckman Coulter, Inc.). Cell gates were designed to select for single and viable mononuclear cells. Defined markers were used to identify the different immune subpopulations. For monocytes, antibodies against CD45, CD3, CD14, CD163 and MHCII were used. To analyze regulatory T cells (T reg) the following antibodies were used: CD45, CD3, TCR γ/δ, CD4, CD8, FOXp3 and CD25, while CD45, CD8, NKp46, CD4, CD8, Ki67 and CD27 were used to identify T and NK cells.
Instrument	Gallios Flow Cytometer (Beckman Coulter, Inc.)
Software	Data were processed using Kaluza 1.5 software (both Beckman Coulter, Inc.)
Cell population abundance	The purity of our targeted population was routinely over 90%
Gating strategy	Gating strategy used for flow cytometric data is presented in Supplementary Figure 7. Firstly, cells were gated on FSC-H vs FSC-W for doublet discrimination. Then, cells were gated on Live/Dead (Dead cell stain, Invitrogen) for identification of live cells. The live leukocytes are assigned as CD45 positive and Live-Dead negative. Plots for specific immune cells population were generated according to the expression of immune markers. For monocytes we used CD3, CD14 and CD163, for T cells gamma/delta T, CD3, CD4 and CD8. Lastly, for natural killers cells we used CD3, CD8 and NKp46.

- [x] Tick this box to confirm that a figure exemplifying the gating strategy is provided in the Supplementary Information.
